# Polymeric Scaffolds Used in Dental Pulp Regeneration by Tissue Engineering Approach

**DOI:** 10.3390/polym15051082

**Published:** 2023-02-21

**Authors:** Vinna K. Sugiaman, Jeffrey, Silvia Naliani, Natallia Pranata, Rudy Djuanda, Rosalina Intan Saputri

**Affiliations:** 1 Department of Oral Biology, Faculty of Dentistry, Maranatha Christian University, Bandung 40164, West Java, Indonesia; 2 Department of Pediatric Dentistry, Faculty of Dentistry, Jenderal Achmad Yani University, Cimahi 40531, West Java, Indonesia; 3 Department of Prosthodontics, Faculty of Dentistry, Maranatha Christian University, Bandung 40164, West Java, Indonesia; 4 Department of Conservative Dentistry and Endodontic, Faculty of Dentistry, Maranatha Christian University, Bandung 40164, West Java, Indonesia; 5 College of Medicine, Veterinary, and Life Sciences, University of Glasgow, Glasgow G12 8QQ, UK; 6 Faculty of Dentistry, Maranatha Christian University, Bandung 40164, West Java, Indonesia

**Keywords:** biocompatible, biodegradable, polymers, scaffolds, tissue engineering

## Abstract

Currently, the challenge in dentistry is to revitalize dental pulp by utilizing tissue engineering technology; thus, a biomaterial is needed to facilitate the process. One of the three essential elements in tissue engineering technology is a scaffold. A scaffold acts as a three-dimensional (3D) framework that provides structural and biological support and creates a good environment for cell activation, communication between cells, and inducing cell organization. Therefore, the selection of a scaffold represents a challenge in regenerative endodontics. A scaffold must be safe, biodegradable, and biocompatible, with low immunogenicity, and must be able to support cell growth. Moreover, it must be supported by adequate scaffold characteristics, which include the level of porosity, pore size, and interconnectivity; these factors ultimately play an essential role in cell behavior and tissue formation. The use of natural or synthetic polymer scaffolds with excellent mechanical properties, such as small pore size and a high surface-to-volume ratio, as a matrix in dental tissue engineering has recently received a lot of attention because it shows great potential with good biological characteristics for cell regeneration. This review describes the latest developments regarding the usage of natural or synthetic scaffold polymers that have the ideal biomaterial properties to facilitate tissue regeneration when combined with stem cells and growth factors in revitalizing dental pulp tissue. The utilization of polymer scaffolds in tissue engineering can help the pulp tissue regeneration process.

## 1. Introduction

Pulpal pathosis is one of the most common oral diseases due to persistent stimulation from trauma, dental caries, or iatrogenic causes. Dental caries occur because of bacterial infection on the tooth surface, which consists of enamel and dentin. Untreated dental caries trigger an inflammation response in the dental pulp, and chronic inflammation in the pulp tissue leads to permanent healthy tissue loss [1,2].

The current pulpal pathosis treatments are root canal treatment and pulp revascularization [2]. Root canal treatment is the treatment of choice in dentistry, which is effective for severe pulpal pathosis conditions. This treatment has a high success rate, but the tooth loses pulp tissue as a result. Thus, despite the treatment’s benefits, the treated tooth becomes nonvital, which increases the risk of fracture and a decrease in the pulp defense mechanism and sensory function [2,3].

Therefore, regenerative endodontic treatment to restore normal pulp functioning via complex dentin–pulp regeneration has recently been developed. The treatment aims to replace the pathological or nonvital pulp tissue with new healthy tissue [2,4].

Regenerative tissue engineering technology is improving rapidly. In pulp tissue regeneration, three important aspects have been developed for their utilization in the technique: stem cells, growth factors, and biomaterials/scaffolds [2,5]. Stem cells represent one of the key elements in tissue engineering technology. Stem cells are unspecialized cells that have the ability to regenerate, proliferate, and differentiate into specific cells [6,7]. After an injury, these cells play a role in healing via tissue regeneration [2,8].

A growth factor or morphogen is a protein or signaling molecule that bonds to specific membrane cell receptors which control and coordinate all cellular functions, such as cell signaling, cell proliferation, and matrix synthesis [6,9]. Growth factors play an important role in increasing the regenerative effect and control function of stem cells. Examples of growth factors that play a role in the signaling process of dentin and pulp regeneration are bone morphogenic proteins (BMP) such as BMP-2, BMP-4, BMP-7, and transforming growth factor β-1(TGF-β1) [4,10,11].

A scaffold or biomaterial is a framework or structure that provides a three-dimensional (3D) growth space for cells and regulates cell function and metabolism. The scaffold creates a microenvironment that promotes cells’ regenerative capacities and multipotentialities. These conditions promote tissue regeneration. Recently, many natural or synthetic scaffold materials have been used for pulp regeneration [2,12]. Bioactive scaffolds stimulate the proliferation and differentiation of stem cells into odontoblast-like cells to regenerate pulp tissue [13,14]. Therefore, the role of scaffolds in tissue regeneration is important, becoming the mediator that facilitates the transfer of stem cells and/or growth factors at the location of the local receptor [15].

Each component in tissue engineering has a different effect in supporting the pulp regenerative process, but a combination of these three components gives the best results [2,4]. Dental tissue engineering is expected to provide tooth vitality, with pulp tissue similar to that of a normal tooth. Therefore, it is important to guide cell interactions with extracellular matrices, which is accomplished by using scaffolds and cell culture techniques [15].

This review will describe the latest developments regarding the usage of natural or synthetic scaffold polymers that have the ideal biomaterial properties to facilitate tissue regeneration when combined with stem cells and growth factors to revitalize dental pulp tissue. The utilization of polymer scaffolds in tissue engineering can help the pulp tissue regeneration process. This article is the first to discuss the various types of scaffolds with their various advantages and disadvantages that can be utilized in regenerating dental pulp tissue.

## 2. The Dental Pulp

Dental pulp is a loose connective tissue that occupies the root canal and is surrounded by dentin. Dental pulp consists of blood vessels, nerves, and odontoblasts, which line the predentine layer in the pulp tissue. Thus, pulp plays a role in providing nutrition, vitality, and pathogen detection through its sensory function as an infection response. Pulp tissue has sensitivity and immunoprotective attributes that maintain pulp homeostasis, facilitate its regenerative ability, and form reactionary dentin [2,16,17,18].

Histologically, dental pulp consists of several zones: the dentinoblastic zone, the cell-free zone, the cell-rich zone, and the pulp core. The primary cells of the pulp layer are odontoblasts, fibroblasts, macrophages, undifferentiated ecto-mesenchymal cells, and other immunocompetent cells [19,20]. The dentinoblastic zone functionally forms the pulp–dentin complex. This zone is the first line of reparative dentine formation and provides protective responses toward external stimulation, whereas the pulp core is rich in nerves and blood vessels which provide the pulp with nutrition and sensory functioning [2,19].

Therefore, the loss of pulp tissue causes a loss of vitality and sensitivity in the tooth and leads to uncontrolled infections in the surrounding tissues. This condition needs complex treatment, such as root canal treatment, which renders the tooth nonvital and brittle, which influences the patient’s quality of life [17,18].

## 3. Dental Pulp Regeneration

Pulp regeneration is a healing process regarding the injured or lost parts of the dental pulp and results in the re-establishment of its complete biological function [2,21]. Ideal pulp regeneration should generate pulp structure and function as similar as possible to healthy tissue. This regeneration involves the regeneration of the dentin–pulp complex, blood vessels, and nerves, which reach a favorable level of reconstruction through the angiogenesis and neurogenesis processes. Other than that, it also involves the rehabilitation of pulp physiological functioning, represented by sensation, nutrition, and immunological defense [2,6].

Illustrated by the formation of connective tissue, with cell density and an architecture similar to that of healthy pulp, successful pulp regeneration consists of nerves and blood vessels able to secrete new dentin as healthy pulp at a controlled rate. Vascular tissue plays a role in providing nutrition, oxygen, cell immunity, and the recruitment and circulation of cells, which maintains the tissue’s vitality and viability, while the nerves are fundamental to cell regulation, which manages the regeneration process and provides defense mechanisms and tissue repair [6,22].

Regenerated blood vessels should be connected to the periapical bone tissue, which surrounds the tooth; therefore, it can receive regular blood flow and transport nutrition for regenerating the tissue or dentin. Other than that, the regenerating tissue should be innervated, with the tooth maintaining heat/cold and pain sensations [17,23]. Therefore, vascular and nerve supply should be maintained through the apical foramen, which is one of the aims of the pulp regenerative process.

In the regeneration process, stem cells proliferate and differentiate into endothelial cells for angiogenesis/vasculogenesis and move into odontoblasts to carry out the dentin reparative process. At the beginning of the process, angiogenic signals, such as fibroblast growth factor (bFGF), vascular endothelial growth factor (VEGF), and transforming growth factor β (TGF-β), are released by endothelial cells, injured pulp cells, and the extracellular matrix (ECM), which causes stem cell migration and stimulates neo-angiogenesis [24,25].

## 4. Endodontic Regeneration

Infected dental pulp needs root canal treatment (RCT), which is a conservative but effective treatment. Traditionally, in this treatment, the pulp tissue is removed and replaced by synthetic obturation materials, such as paste or gutta-percha [13,17]. RCT aims to remove the space for potential microbiome reinfection and create a healing environment by mechanical or chemical disinfection, which is continued by inert material closure [2,26]. The treatment has a high success rate in dentistry, with 97% of one million teeth able to retain functionality for around 8 years [13,17].

Teeth that receive RCT experience severe defects regarding hard tissue, devitalized pulp from denervation, and avascularity. This leads to an increased risk of fracture, the disruption of the pulpal defense mechanisms, and a loss of physiological functions, such as nutrition and sensation [2,17,27]. In order to prevent these side effects, an effective treatment strategy is needed for the revitalization of the pulp. The emergence of tissue engineering technology and regenerative treatments provides the possibility of developing regenerative endodontic treatments [17].

RCT causes the tooth to be nonvital and susceptible to structural changes [28]; the challenge in modern dentistry is to maintain pulp vitality. Thus, an interdisciplinary approach to regenerative treatments has developed, which utilizes living cells to heal, replace, and restore damaged human tissues and organs to reach their normal level of functioning. One of these treatments is stem cell engineering, which has the potential to be the future of regenerative treatment [29,30].

Dental tissue regeneration can be obtained by the regeneration of each part of a tooth’s structure, which consists of enamel, dentin, pulp, alveolar bone, cementum, and periodontal ligament or by regenerating the whole tooth structurally and functionally [15,31]. Regenerative endodontics is one of the endodontic treatments that focus on replacing the damaged pulp tissue through tissue regeneration to restore tooth vitality, leading to an increase in patient quality of life. Regenerative tissue should have healthy pulp properties, such as the ability of the dentin-deposition process, reinnervation, and vascularization [17,26].

## 5. Tissue Engineering

Tissue engineering technology is an interdisciplinary science that implements the biological principles of regenerative treatment techniques, with a focus on repairing and restoring the biological function of cells, tissues, and organs that have been injured by internal or external factors [6,32]. Tissue engineering technology aims to contribute to the restoration of damaged tissue function and structure by utilizing stem cell interactions, scaffolds/biomaterials, and growth factors. The proper combination of these three elements enables the manipulation of the biomimetic microenvironment containing the vascular system, which normally maintains nutrition supply, waste disposal, inflammatory response, and pulp regeneration [2,6,33]. In tissue engineering, angiogenesis has an important role in nutrition supply and the potential recruitment of stem cells [4,34].

In tissue engineering technology, pulp regeneration might be achieved via the utilization of three key elements: (i) stem cells, (ii) scaffolds, and (iii) signaling molecules such as growth factors. Firstly, the pulp regeneration process might be achieved through stem cell isolation and in vitro manipulation. After this, the cells are cultured in the scaffold and combined with the growth factor, which is then all transplanted into the root canal [35,36,37].

Every individual element has a different impact on pulp regeneration, but with all elements supporting each other, this might provide a favorable result. The proper combination of these three elements provides a micro-biomimetic environment, influencing the overall accomplishment of pulp regeneration. This result might be achieved by the formation of a fully functional vascular system, thus providing adequate nutrition supply, waste disposal, and inflammation response, leading to satisfactory pulp regeneration [2].

### 5.1. Dental Stem Cells

Mesenchymal stem cells (MSCs) are a type of stem cell that is suitable for regenerative treatment because of its high proliferation and multipotential ability [29,38]. According to the minimal criteria of the International Society for Cellular Therapy, MSCs are marked with positive (CD29, CD44, CD73, CD90, CD105, and Stro-1) and with negative hematopoietic markers (CD14, CD34, and CD45) [13,39].

MSCs can be isolated from different locations in the oral and maxillofacial regions, such as from dental pulp stem cells (DPSCs) and the stem cells exfoliated from human deciduous teeth (SHED) and can be isolated from healthy pulp tissue. These cells could be differentiated in vitro into adipocytes, odontoblasts, osteoblasts, and chondroblasts, which form dentin or pulp tissue after in vivo transplantation [13,29]. Other cells, such as dental follicle progenitor stem cells (DFPCs), periodontal ligament stem cells (PDLSCs), and stem cells from apical papilla (SCAPs), can be differentiated in vitro into adipocytes, odontoblasts, cementoblast-like cells, and connective tissue [5,13,29,40].

Each type of stem cell has different properties: SHED and SCAP have higher proliferation activity compared to DPSC, although all stem cells possess the potential to regenerate dentin and pulp [5,13].

### 5.2. Growth Factors

Signaling molecules, such as stem cell factor (SCF), stromal-cell-derived factor (SDF-1α), platelet-derived growth factor (PDGF), basic fibroblast growth factor (bFGF), and granulocyte colony-stimulating factor (G-CSF), can be used for pulp tissue regeneration [17]. Several growth factors, such as SDF-1α, bFGF, and PDGF, are chemotaxis molecules and correlate to blood vessels, nerves, and dentin in the pulp regeneration process. PDGF and VEGF contribute to vasculogenesis/angiogenesis, while NGF contributes to the growth and survival of the nerves; BMP-7 contributes to the differentiation and mineralization of odontoblasts [36,37]. Growth factors play a role in the restoration of stimulation of a structure and the physiology of tissue function in damaged tissue [2].

### 5.3. Scaffolds

A scaffold is a three-dimensional frame microenvironment that facilitates attachment, cellular infiltration, differentiation, proliferation, and stem cell metabolism with the aid of growth factors. The frame has to provide support for nutrition and oxygen diffusion in the regeneration process and should have biodegradable properties because it will be replaced by the new tissue [4,6,41].

Different types of developed scaffold materials or models have certain levels of flexibility and degradability [6]. Currently, natural or synthetic scaffolds have started to be commonly used in pulp tissue regeneration [2]. The scaffolds that have been used are tissue extracts, such as blood clots, platelet-rich fibrin (PRF), platelet-rich plasma (PRP), tricalcium phosphate ceramic, hydroxyapatite calcium, and mineral trioxide aggregate and synthetic polymers such as polylactic-co-glycolic acid, polylactic acid, and biopolymers such as collagen, hydrogel, hyaluronan, and chitosan [4].

Blood clots represent one type of scaffold that has natural properties from which natural substances such as collagen, chitosan, fibrin, hyaluronic acid, gelatin, alginate, and peptide-based scaffolds can be derived. These scaffolds have been studied as scaffolds for pulp regeneration because of their biocompatibility, biomimetic properties, availability, cost-effectiveness, and ease of conversion (into hydrogel) [13,42].

Other than natural scaffolds, there have been several synthetic polymers developed, such as polyglycolic acid (PGA), poly(d,l-lactide-coglycolide) (PLGA), polylactic acid (PLA), poly(l-lactic) acid (PLLA), and polycaprolactone (PCL), and inorganic calcium phosphates, such as hydroxyapatite (HA) or beta-tricalcium phosphate (β TCP), as well as a combination of silica glass and phosphate. Synthetic scaffolds have been studied considerably as scaffolds that have the potential for tooth regeneration because of their nontoxicity, biodegradability, and ease with which to manipulate properties, including mechanical rigidity and degradation rate [2,15,42].

In contrast to natural scaffolds, synthetic scaffolds can be prepared in unlimited numbers because they are produced in a controlled environment according to a desirable shape. This condition allows for the obtainment of the scaffold in accordance with cell differentiation properties, certain pore characteristics, and certain mechanical, chemical, and degradation rate properties according to the desired application [15,43,44].

This polymer is a biomaterial that is commonly used to form scaffolds with characteristics that are related to differentiation in their composition, structure, and macromolecule arrangement [15]. In recent studies, scaffolds have shown the potential to be bioactive carriers and have recapitulated the interaction between stem cells, progenitor cells, micro-physiological environments, and extracellular matrices [13]. In regenerative endodontic treatment, polymer scaffold usage could provide physiological environments to increase the biological performance in the pulp regeneration process. This process consists of revascularization and revitalization processes. This scaffold influences cell migration, viability, discharge, proliferation, recruitment, and degradability [45].

Although scaffolds have huge potential, there are challenges that need to be overcome, such as integrating the scaffold with complicated morphologies without damaging the surrounding tissues. For tooth regeneration, scaffolds require several general characteristics, such as being easy to manipulate, having bioactive and biodegradable properties, having adequate porosity and physical and mechanical strength, having low immunogenicity, and being able to support vascularization [15,43].

Other criteria, such as having an adequate shape, size, and pore volume, are important for the penetration and diffusion of growth factors, nutrition, and waste discharge between the cells [13,15]. Therefore, a scaffold’s criteria and design create favorable microenvironments that are important as a foundation to then perform tissue engineering technology processes. This microenvironment supports the organization of cell functioning regarding self-renewal and differentiation, supporting cell and growth factor transportation, creating an environment for cell activities, and promoting communication between cells, which leads to tissue regeneration [2,13,46]. These scaffold characteristics represent important keys to the process of tissue regeneration because they play vital roles in defining cell behavior and tissue formation [13].

**Figure 1:**
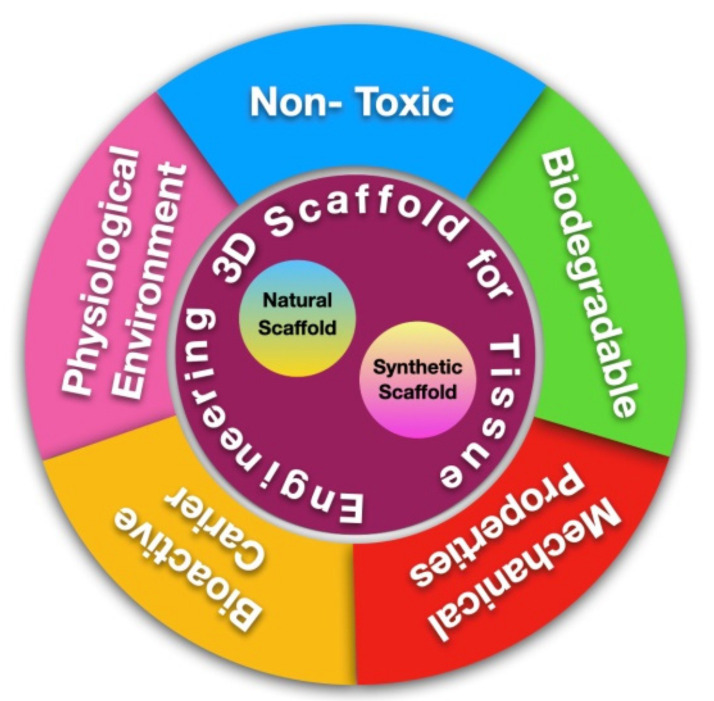
Scaffold for Tissue Engineering.

To confirm the success of the cell growth and differentiation processes in tissue engineering, scaffold materials must be able to interact with host tissues and provide an ideal environment for tissue growth [29,46]. The ideal scaffold for pulp regeneration should fulfill three criteria: biocompatibility, adequate rigidness to withstand mastication force, and tight sealing with dentin to prevent micro-organism infiltration [29,44]. Other than that, the degradation process of a scaffold is usually one of the factors that plays a role in treatment failure [47]. The rate of scaffold degradation should be complementary to the rate of new tissue formation and should not produce harmful waste side products [15,48,49]. Utilization of the use of scaffolds in tissue engineering technology must fulfill several characteristics which can be seen in Figure 1.

#### 5.3.1. Scaffolds Made of Natural Polymers

One of the tissue engineering triad elements in regenerative endodontics is scaffolds, which work as biological and structural support for cell growth and differentiation. Proper scaffold selection is a challenge in the dentin–pulp regeneration process [50]. Cells’ migration, proliferation, and differentiation correlate with the choice of a scaffold’s physical properties, such as appropriate viscoelasticity to mimic the real pulp tissue [51]. The application of scaffolds for dental pulp regeneration should be able to mimic the microenvironment in the root canal and provide mechanical support [52,53].

The application of 3D bioprinting technology to scaffold-making can precisely mimic external and internal morphologies. The 3D scaffold has moderate porosity, which allows nutrition and oxygen infiltration, leading to the occurrence of metabolic activities [53]. The application of scaffolds via the injection process is recommended because it can adapt well to the shape of the pulp chamber and root canal so that cell and matrix interaction can occur efficiently [50].

To date, scaffolds are classified as natural and synthetic scaffolds based on the material source and biomaterial properties used [54]. Scaffolds for tissue regeneration using natural or synthetic materials are continually being developed [55]. Natural scaffolds come from the host or natural materials. Examples of host scaffolds are blood clots, autologous platelet concentrates, and decellularized extracellular matrices [54]. Examples of natural material scaffolds are collagen, alginate, chitosan, hyaluronic acid, and fibrin [50,51,53,54]. Natural material scaffolds have the advantage of cell recognition and adhesion from molecular signaling, although the application of this type of scaffold has the limitation of product variation, risk of pathogen transmission, poor mechanical properties, and immunological responses to foreign objects [52]. The shape of the scaffold can be a porous sponge, a solid block, a sheet, or a hydrogel [56].

Collagen is a scaffold material that has the closest viscoelasticity to real pulp tissue [51]. The combination of natural materials, such as collagen and the host’s blood clot, show predictable patterns for tissue formation and mineralization in human dental structures when compared to collagen or blood clots individually. The application of one type of scaffold, such as a blood clot, does not provide stable results for the tissue regeneration process [57]. Instability and unpredictable clinical results from the blood clot are the consequences of unregulated stem cells in the pulp chambers, including the difficulties of bleed formation and hemostasis [52].

When compared to blood clots, platelet-rich plasma (PRP) and platelet-rich fibrin (PRF) provided lower increases in dental root length and less effectivity in root development [58]. PRP from the host’s blood contains high platelet, growth factor, and cytokine concentrations, which increase the ability of wound healing and stem cell recruitment from the pulp and increase SCAP proliferation. While PRF contains plentiful growth factors, which can stimulate cell differentiation as well as cell adhesion and migration [59]. The advantages of materials with rich platelet concentrations, such as PRF or PRP, are the increases in the level of angiogenesis and revascularization, which is fundamental to accomplishing endodontic regeneration therapy [56]. Hydrogel-based collagen could mimic the interaction between cells and extracellular matrices in vivo and organize cell growth, which is used for tissue engineering [60].

Polymer materials, such as gelatin and fibrin, are commonly used as natural scaffolds. Gelatin is a biopolymer protein that comes from collagen hydrolysis, which facilitates the proliferation and differentiation of odontoblasts in dental pulp stem cells (DPSCs) [50]. Gelatin is a partial hydrolysate from animals. When compared to gelatin, hydrogel gelatin has better biocompatibility because of its low immunogenicity properties [50,53]. A gelatin-based matrix showed better endodontic therapy results when compared to fibrin-based matrix groups after 12 weeks follow-up in mini-pig immature dental models [50].

Other studies into fibrin-based scaffolds in hydrogel showed that this material was compatible with dental pulp regeneration by supporting pulp-like tissue formation [61]. Fibrin is a natural protein polymer that forms part of blood clot formation. Hydrogel-based fibrin can stimulate pulp-like tissue formation with an odontoblast layer in the root canal system [50]. The advantages of these materials are good cytocompatibility, physical kinetic degradation, and nontoxic degradation products, and they are also easy to inject into the pulp canal. Other natural materials, such as alginate, chitosan, collagen, and hyaluronic acid, or synthetic materials, such as polyethylene glycol, poly (D,L) lactic acid, and fibrin-based bio-ink for 3D printing, were added to increase the structural and functional properties of fibrin scaffolds [61].

Alginate is a natural polymer from algae, which has good biocompatibility properties, is cost-effective, has low cytotoxicity, and has an optimal structure for nutrition exchange [45,52,53]. Alginate hydrogels were formed by crosslinking polysaccharide and divalent cations to form an ion bridge in water-insoluble tissue [52]. Alginate hydrogels are able to arrange themselves in accordance with mechanical properties, such as rigidity and stress relaxation, to regulate stem cell activity [45]. Alginate has proper mechanical properties but can be applied in the form of hydrogel injection or bone porosity, which enables the natural structure to be loaded with growth factor [56] The macroporosity of alginate scaffolds enables the exchange between nutrition and metabolism waste. However, scaffolds that consist of only alginate have a limited role in endodontic regenerative therapy; therefore, its combination with other materials, such as bioactive polymers, is needed [52].

Hyaluronic acid (HA) is a biopolymer that can be modified and processed for biomedical applications, and it can be combined with other materials to increase its favorable properties [60]. HA in dental pulp was found to decrease dental development in the odontogenesis process [52]. When applied to exposed pulp, HA can stimulate the production of reparative dentin. HA can be applied in 3D-sponge form to create a proper environment for blood vessel proliferation and stem cell differentiation [56]. HA is formed by d-glucuronic acid and N-acetyl-D-glucosamine and is commonly available in the form of liquid injection [45]. HA degradation products include pro-angiogenic growth factors, which represent the revascularization elements of dental regeneration tissue, although HA has the disadvantages of poor mechanical properties and can cause hypersensitivity reactions [52].

Chitosan is a widely used natural scaffold [62]. Chitosan is a cation polymer from chitin [55] Chitosan has good biocompatibility, biodegradation, and other favorable biological properties, such as being antimicrobial, fungistatic, and noncarcinogenic, with hemostatic and protein fusion abilities, as well as being able to stimulate cell adhesion, proliferation, and differentiation [55,62]. However, the application of chitosan is difficult because of the complex gelation and degradation process due to unusual polycationics and a highly crystalline structure, which limits the application of this type of scaffold to the form of a natural injection [52]. The hydrogel form of chitosan can be injected into the dental pulp chamber [62]. Chitosan can be applied as an individual scaffold or in combination with polymers or other biomaterials to produce a large number of matrices for tissue engineering purposes. The addition of chitosan scaffolds into the blood for endodontic regeneration procedures can stimulate the formation of new soft tissue (as proven by histological regeneration) without the formation of mineralized tissue around the pulp canal wall [55]. Additional photo-biomodulation therapy could increase in vitro stem cell survival, proliferation, and migration from the root papilla [62].

When comparing several natural scaffolds, other studies have shown that human teeth can be applied as scaffolds for periodontal ligament and pulp regeneration [26]. Scaffolds from natural materials have higher biocompatibility and bioactivity properties when compared to synthetic scaffolds, whereas synthetic scaffolds have higher controlled degradation levels and mechanical properties [63]. The application of scaffolds that are not limited to the use of only one material, i.e., those that can be combined, can provide better endodontic regeneration therapy.

#### 5.3.2. Scaffolds Made of Synthetic Polymers

The implantation of 3D scaffolds in the appropriate living cells that secrete their own extracellular matrix (ECM) can provide an acceptable environment. The adequate porosity and permeability of a polymeric scaffold are essential for guiding and supporting the cultured cells’ ability to produce tissue. Synthesizing synthetic biodegradable polymers is challenging in tissue engineering applications [64,65].

The progenitor/stem cells should then be able to attach, travel through, proliferate, and organize themselves spatially in 3D space and differentiate into odontogenic, vasculogenic, and neurogenic lineages with the support of an adequate scaffold for dentin–pulp regeneration. Furthermore, the biocompatibility of the material is critical to avoid any negative reactions from the host tissue. Biodegradability that can be adjusted to match the rate of regeneration is critical for facilitating constructive remodeling. As a result of scaffold deterioration, a series of tissue responses occur, comprising the targeted tissue replacement of the scaffold, vascularization, differentiation, spatial structure, and cellular infiltration [66,67,68].

Metals, ceramics, and polymers are examples of materials that can be used to make scaffolds. Both dental and bone implants are frequently made of metallic alloys. When it comes to bone tissue engineering, ceramics with strong osteoconductivity have been used, although metals and ceramics have substantial disadvantages because metals do not biodegrade and do not serve as a matrix that mimics biological processes for the proliferation of cells and tissue creation. Additionally, due to brittleness, ceramics are difficult to convert into highly porous structures and have a limited capacity for biodegradation. In contrast, polymers can be molecularly designed to have increased biodegradability and excellent processing flexibility. Therefore, for tissue engineering, polymers are the most common type of scaffolding material [31,68,69,70].

Biological recognition represents one potential benefit of naturally generated polymers, which may help to stabilize cell adherence and ensure proper function. The synthetic polymers used as scaffolding materials have been spurred on by the challenges associated with natural polymeric materials, such as their complex purification, structural composition, pathogen transmission, and immunogenicity. When compared to naturally occurring extracellular matrix (ECM) proteins, synthetic polymers offer better processing flexibility and no immunological issues. Functionalized scaffolds that combine the benefits of synthetic and natural polymeric materials can be made by adding bioactive molecules to synthetic polymers [69,70,71].

The advantages of synthetic polymers include nontoxicity, biodegradability, and the ability to precisely manipulate their physicochemical characteristics, such as degradation rate, structural rigidity, microstructure, and porosity [72,73,74]. Natural polymers are mostly broken down by enzymes, but synthetic polymers are typically broken down by simple hydrolysis. However, because of the relative acidity of the hydrolytically destroyed byproducts, synthetic polymers might cause localized pH reductions and a chronic or acute inflammatory host response [74,75,76].

Tissue engineering frequently uses poly (-hydroxy acids), such as poly (lactic acid), poly (l-lactic acid), poly (glycolic acid), polyethylene glycol, and their copolymers poly [(lactic acid)-co-(glycolic acid)] (PLGA) and poly-epsilon caprolactone (PCL), which appears to be the most synthetic polymeric material. These polymers have an established track record and have been approved by the FDA for specific human applications (e.g., sutures). Two of the synthetic polymer scaffolds that have been suggested for dental tissue engineering are PGA and PLA, which are biodegradable polyesters that can be produced from a range of renewable sources. When compared to PGA, PLA, which is an aliphatic polyester, is more hydrophobic [66,69,74,76,77,78].

The synthetic scaffold known as PGA, which has been used for cell transplantation, breaks down when the cells secrete an ECM. Several cell types, including cellular origins of dental pulp, pulpal fibroblasts, and ex vivo human pulp tissue cells, have been shown to be able to adhere and develop on PGA scaffolds. The copolymers of PGA and PLA that are sown with dental pulp progenitor cells have been shown in rabbit and mouse xenograft models to produce pulp-like tissue [66,69,74,75].

Since structural strength is vital in many applications, PLLA, an extremely strong polymer, has been used in several of them. Nanofibrous scaffolds have been created from it that resemble the structure of genuine collagen (a crucial element of ECM). It has been shown that nanofiber PLLA scaffolds promote cell attachment and differentiation. Previous studies demonstrated how PLLA scaffolds could stimulate the development of endothelial cells from dental pulp cells and odontoblasts [66,69,75]. This was demonstrated by utilizing PLGA as a scaffold from which dentin-like tissue could emerge and in which pulp-like tissue could be repaired over the course of 3 to 4 months. A 50:50 blend of PLGA degrades after around 8 weeks. PCL, a slowly disintegrating polymer, has been utilized in bone tissue engineering projects either by itself or in conjunction with hydroxyapatite [75].

A different type of polymer, polyethylene glycol, is utilized in tissue engineering techniques, such as pulp regeneration. Dental pulp progenitor cells have been transformed to create 3D-tissue constructs while being linked to electrospun polyethylene glycol scaffolds. These artificial polymer scaffolds have also been utilized to convey a range of substances, including anti-inflammatory drugs, growth hormones, and sticky proteins. Such scaffolds could not only support cell growth and proliferation but could also reduce pulpitis and aid in pulpal healing. Synthetic polymer scaffolds have better handling characteristics and a more straightforward manufacturing process, which improves their potential for endodontic regeneration. They do, nevertheless, differ significantly from the natural dental pulp extracellular environment. As a result, ECM-based natural scaffolds that are closer to the microenvironment have been developed [66,74,79,80].

Planting human exfoliated deciduous teeth stem cells (SHED) on dentin disks with PLA resulted in the structure of odontoblast-like cells, new dentin, and vascularized pulp-like tissue. A study by Huang et al. illustrated that when implanted in vivo into an empty root canal area, the stem cell constructions made from the apical papilla (SCAPs) and L-lactide, poly-D, and glycoside were able to create soft tissue that resembles pulp, with the continual addition of new dentin to the surface. However, synthetic polymers have the potential to cause an immediate or long-lasting inflammatory response. Additionally, the locally decreased pH brought on by the hydrolytically degraded metabolites may impair its clinical use [66,75].

Several methods have been used to construct 3D scaffolds from poly (hydroxy acids). The inability of the poly (a-hydroxy acids) chains to allow functional groups, however, restricts the incorporation of biologically active moieties onto the scaffolding surface. In order to increase the functioning of these polymers and broaden their usage, significant efforts have been made in this direction; creating copolymers out of a-hydroxy acids with additional monomers that have functional pendant groups, including amino and carboxyl groups, is one technique. In one study, ring-opening polymerization was used to copolymerize (RS)-b-benzyl malate and L-lactide; then, the benzyl groups were removed to create (RS)-b-malic acid) poly (L-lactide) with connected carbonyl compounds [69,81,82].

In order to copolymerize this with L-lactide, benzyloxymethyl methyl glycolide and benzyloxymethyl glycolide are required, which have preserved hydroxyl groups. The matching hydroxylated PLLA copolymers were produced when the benzyloxymethyl groups were unprotected. Comparable carboxylic acid functionalized copolymers can be created using succinic anhydride [69,83].

The researchers created a poly [(L-lactic acid)-co-(L-lysine)] containing a useful lysine residue that they further linked to the RGD peptide. Even though the development of functional groups in random copolymers by lactide/glycolide copolymerization with additional monomers can be successful, this procedure frequently affects the physical characteristics of the starting homopolymers, such as crystallinity and mechanical strength. Numerous block and graft copolymers based on poly(a-hydroxy acid) have been developed and made as a result of this [69,84].

Polymer PEG, or poly (ethylene glycol), is the component that is most frequently used in (a-hydroxy acids). PL(G)A/PEG diblock, triblock, and multiblock copolymers could be made by the ring-opening of PEG and certain catalysts and the presence of glycolide/lactide polymers. However, the hydroxyl or carboxyl (functional groups) in the block copolymers containing PEG are only present at the end of each PEG segment, and the content in these block copolymers is very low, further restricting chemical alterations. Numerous block and graft copolymers made without PEG have been described [69,85].

Amphiphilic poly [hydroxyalkyl (meth) acrylate)] is a variety of biodegradable polymer. Copolymers of -graft-poly (L-lactic acid) (PHAA-gPLLA) with hanging hydroxyl groups were employed to successfully produce 3D-nanofibrous scaffolds. The further functionalization of these copolymers can result in biomimetic scaffolds that are more hydrophilic, degrade more quickly, and have uses in tissue engineering [69,86].

The fabrication of highly porous poly (α-hydroxy acid) scaffolds can be used for tissue engineering based on star-shaped functional poly(ε-caprolactone). The functional groups were added to PCL chains using similar methods. Examples of these methods include the copolymerization of ε-caprolactone and a-chloro-ε-caprolactone to produce functionalized PCL copolymers, and the subsequent addition of carboxyl, pendant hydroxyl, and epoxide groups via atom transfer radical addition. In order to produce the pendant hydroxyl groups in the PCL copolymers, ε-caprolactone was copolymerized with another monomer, 5-ethyleneketal-ε-caprolactone, and the resulting molecule was subsequently deacetylated to convert the ketone groups into hydroxyl groups [69,87].

However, these deprotection processes (as well as the synthesis of these functional comonomers) are typically challenging and time-consuming. Aside from poly (3-hydroxybutyrate), polyurethanes, polycarbonate, poly (ortho ester), poly (propylene fumarate), and polyphosphazenes, other synthetic biodegradable polymers have also been used as scaffolding biomaterials. Comparatively, there are many fewer reports of the functionalization of these biomaterials (a-hydroxy acids), which include the creation of functionalized PC using synthetic methods [69,87,88].

Pendant amino groups were added to PC chains after polymerizing the cyclic carbonate monomer (2-oxo-[1,3]-dioxan-5-yl) carbamic acid benzyl ester and disposing of the protective benzyloxy carbonyl groups. The pendant amino groups’ further functionalization was shown using RGD peptide grafting; synthetic efficiency should be considered, given the number of steps in this reaction cycle [69,89].

The five distinctive structural characteristics of these PAs are as follows: (1) an extended alkyl tail that contributes to the molecule’s amphiphilic characteristic; (2) maintenance of the structure by possessing four consecutive cysteine residues that create disulfide bonds; (3) a flexible hydrophilic head group due to the three glycine residues in the linker region, which separates the hard cross-linked region; (4) phosphorylated serine residues that interact strongly with calcium ions to encourage mineralization; and (5) an effective RGD peptide [69].

The high electrostatic interaction between molecules causes the PAs to self-assemble into nanofibrous networks when the pH is changed or when divalent ions are added, as evidenced by this study. Additionally, the hydrophilic peptide signals can be displayed in a specific way on the surfaces of the produced nanostructures due to the molecule’s amphiphilic characteristics. However, the creation of sufficient mechanical three-dimensional structures from these PAs must be addressed, as is true for several other hydrogel materials. Proteinase-sensitive motifs represent an inventive technique to make biomaterials react to cells [69,90].

As cell-ingrowth frameworks for tissue formation, Hubbell et al. presented a valuable example of how to build synthetic PEG-based hydrogels. The functionalization molecules for PEG chains in hydrogel networks, which also include pendant oligo peptides (RGDSP) for cell attachment, are matrix metalloproteinase (MMP)-sensitive peptides. The material’s reaction to the MMPs secreted by cells is controlled by the MMP-sensitive binding agent. This hydrogel, with a PEG foundation, functions as a biomaterial and reacts to cells. The authors also showed that these gels could promote bone regeneration and are efficient delivery systems for recombinant human bone morphogenetic protein-2 (rhBMP-2) [49,69].

Many of the requirements for the dental pulp tissue engineering approach may be accommodated by self-assembling, adaptable, and customizable peptides. Due to the peptide chains’ natural amino acid makeup, they can produce biodegradable products. The potential for uniform cell encapsulation, the rapid transport of nutrients and metabolites, and the characteristics of peptide hydrogel systems are affected by their viscoelastic properties, which are comparable to the properties of collagenous tissues such as dental pulp [66,91].

The term “bioceramic scaffolds” refers to a group of materials, including glass ceramics, bioactive glasses, and calcium/phosphate compounds. Calcium phosphate-based (CaP) ceramics are the biomaterials that are utilized most frequently. Due to their characteristics of osteoclast genesis, nontoxicity, antigenicity, osteoinduction, bone bonding, and similarity to mineralized tissues, CaP scaffolds, such as -TCP or HA, have been extensively explored for bone regeneration. Three-dimensional CaP porous granules have demonstrated their potential in the engineering of dental tissue by providing excellent 3D-substrate characteristics for hDPSC growth and odontogenic differentiation. Pure TCP scaffolds are doped with SiO2 and ZnO to increase their mechanical stability and capacity for cellular proliferation. Glass ceramics made of SiO2 Na2OCaOP2O5 are bioactive and offer ideal crystallization conditions. The osteoblastic activity of the substance is increased by the release of dissolving products, such CaP [15,75]. 

Ceramic scaffolds can be altered to control the dissolving rate, provide the appropriate permeability, and control certain surface properties to promote cellular activity. The mechanical rigidity of the scaffold is influenced by variations in pore size and volume. Glass ceramics made of magnesium can increase mechanical strength and provide a high rate of bioactivity. Excellent hDPSC attachment, proliferation, and differentiation have been demonstrated by niobium-doped fluorapatite glass ceramics [75,92].

The several disadvantages of bioceramics include a longer creation time, the lack of an organic phase, nonhomogeneous particle size and form, huge grains, difficulty to shape, brittleness, slow degradation, and high density. Bioceramics are fragile and have little mechanical strength when individually utilized. This drawback can be remedied by combining them with polymer scaffolds [75,92]. Comparison of various types of scaffolds for tissue engineering can be seen in Table 1.

Tissue engineering technology requires a scaffold as a porous structure that can assist in tissue regeneration. In addition to various scaffold properties with various advantages needed to provide mechanical support in the regeneration process, tortuosity is also an important parameter in developing the permeability of 3D scaffolds to be used in tissue engineering technology. This affects the occurrence of cell attachment, proliferation, differentiation, and cell migration in the process of tissue regeneration [101,102].

Research on tissue engineering technology has not been widely carried out in humans, so this study cannot discuss how far its success has been when applied to living tissue. Therefore, the application of various types of polymer scaffolds needs to be developed further.

## 6. Conclusions

Various types of scaffolds, both natural and synthetic, can be used to regenerate dental pulp by utilizing tissue engineering technology. Scaffolds made from natural materials have advantages in cell recognition and molecular signal adhesion, while synthetic scaffolds can be made in unlimited quantities. However, a better effect might be realized if the two types of scaffolds are combined to obtain good mechanical properties so that they can support pulp regeneration properly. In the future, it is hoped that more extensive research can be carried out on various types of scaffolds so that not only polymer-based scaffolds are described for the regeneration of dental pulp tissue.

## Figures and Tables

**Table 1:** Comparison of various types of scaffolds for tissue engineering.

Article (Author, Year)	Type Scaffold	Properties	Advantages	Disadvantages
Alaribe, 2016; Jang, 2020; Ducret, 2021 [50,61,93]	Fibrin	Biodegradation, protein natural blood clot, hydrogel base, stimulates the formation of odontoblast	High adhesion to surface, good cytocompatibility and biodegradability, nontoxic, easy to inject	Produced by the body after an injury
Alaribe, 2016; Palma, 2017; Moreira, 2021; Raddal, 2019 [52,55,62,93]	Chitosan	Easier to process, hydrogels, films, fibers or sponges, gel-forming abilities; chitosan hydrogels: low viscosity, high adsorption capability. Chitosan, which is the cationic polymer of chitin, has the attractive properties of biodegradability.	It has been used extensively, can support the differentiation of stem cells, noncytotoxicity, biocompatible, biodegradable, antitumor, antifungal, antibacterial activity, nonimmunogenicity, Easily processes, enhances proliferation and cell attachment, hemostatic, noncarcinogenic	Hard use; high crystalline structure: limited application
Amini, 2021; Erisken, 2015; Nosrat, 2019; Ayala-Ham, 2021; Raddal, 2019; Liu, 2022 [51,52,54,57,60,73]	Collagen	It lacks structural stability, good mechanical properties, and a material that is comparable to soft dental pulp’s viscoelastic properties, more recommended in combination with a blood clot, hydrogel-based: mimics interactions between cells and ECM in vivo, type 1 collagen is most used.	Low antigenicity, high biocompatibility; biodegradability, bioactivity, and good cell adhesion, high mechanical strength, the ability to cross-link	Problems with controlling space and the rate of degradation, as well as difficulties with sterilization and processing, pathogen transmission low mechanical properties, irregular biodegradation, risks immunogenicity
Amini, 2021; Wu, 2021; Raddal, 2019; Yu, 2019; Nowicka, 2021 [45,52,53,56,73]	Alginate	Requires a multistep purification procedure to achieve extremely high purity, natural polymer from algae; alginate hydrogels: crosslinking polysaccharides and divalent cations, the mechanical properties can be adjusted (alginate hydrogels)	High biocompatibility and biodegradability, low toxicity, chelating properties, and non-antigenicity, cheap price, low toxicity optimal structure for exchange nutrition	Endotoxins, heavy metals, polyphenolic and protein compounds, as well as compounds derived from marine sources, are among the naturally occurring impurities; poor mechanical properties. It must be combined with other polymers.
Amini, 2021; Ayala, 2021; Raddal, 2019; Wu, 2021; Nowicka, 2021 [45,52,56,60,73]	Hyaluronic Acid	Nanofibrous scaffolds, water insolubility, modified biopolymer; in dental pulp, the amount decreases according to the process of odontogenesis, contains d-glucuronic acid and N-acetyl-D-glucosamine, available in liquid injection form.	Excellent biocompatibility, high water content, suitable viscoelastic properties for many tissue types, capacity to degrade into safe products, and the capability to join to the specific cell surface receptors, reparative dentin stimulation, 3D sponge shape suitable for blood vessel proliferation and stem cell differentiation	It is impossible for the cells to adhere to the surface, low mechanical properties, hypersensitivity reactions, and minor biodegradability
Amini, 2021; Farzamfar, 2017; Gathani KM, 2016; Dissanayaka WL, 2020 [66,73,75,94]	Poly (lactic acid) (PLA)	Good mechanical strength	Biocompatibility, processability, biodegradability; planting human exfoliated deciduous teeth stem cells (SHED) on dentin disks with PLA resulted in the structure of odontoblast-like cells, new dentin, and vascularized pulp-like tissue	Low impact toughness, hydrophobicity, and a slow rate of degradation
Amini, 2021; Gaaz, 2015; Gathani KM, 2016; Dissanayaka WL, 2020; Liu X, 2012 [66,69,73,75,95]	Poly (l-lactic acid) (PLLA)	Excellent porosity, a high surface-to-volume ratio, nanofibers, and a variety of pore-size distributions	Biodegradable, promotes cell attachment and differentiation, PLLA scaffolds encouraged the development of endothelial cells from dental pulp cells and odontoblasts	During degradation, hydrophilicity, biocompatibility, and mechanical properties are all poor
Zhai, 2015; Gathani KM, 2016; Dissanayaka WL, 2020; Liu X, 2012 [66,69,75,96]	Poly (glycolic acid) (PGA)	Highly crystalline and hydrophilic linear polyester, better solubility in water, degradation half-life is about 2 weeks	Help attachment cell	Degradation rate is too high
Barroca, 2018; Amini, 2021; Saini, 2016; Santoro M, 2016; Dissanayaka WL, 2020; Rizk A, 2013; Danhier F, 2012 [66,73,79,80,97,98]	Polyethylene glycol (PEG), Copolymer poly [(lactic acid)-co-(glycolic acid)] (PLGA),	Crystallinity, glass transition temperature, good mechanical Strength	Biodegradable, biocompatible, low toxicity/swelling; these artificial polymer scaffolds have also been utilized to convey a range of substances, including anti-inflammatory drugs, growth hormones, and sticky proteins, and support cell growth and proliferation, reduce pulpitis and aid in pulpal healing	The degradation pattern of PLGA is highly dependent on the sequence of monomers that make up its structure, which liberates acidic products
Mir M, 2017; Amini, 2021; Sisson, 2013; Gathani KM, 2016 [73,75,99,100]	Poly-epsilon caprolactone (PCL)	Good mechanical properties, high elasticity, high strength	Biocompatible, biodegradable, low toxicity, slowly disintegrating polymer, has been utilized in bone tissue engineering projects either by itself or in conjunction with hydroxyapatite	Hydrophobicity, slow degradation, lack of functional groups

## Data Availability

Data sharing not available.
